# Supplementary material for: The discovery of novel noncoding RNAs in 50 bacterial genomes
Source: Nucleic Acids Res. 2024 Apr 22;52(9):5152–65. doi: 10.1093/nar/gkae248 (PMC11109978; doi:10.1093/nar/gkae248)
Supplement: gkae248_Supplemental_Files [file gkae248_supplemental_files.zip › Narunsky 50 Genomes RNA Motifs Files.zip!/Publication/80-C_botulinum/Figures/LRC-80-13-NC_009698_1_IGR_226_2119730_2120188.sto.pdf]

LRC-80-13-NC_009698_1_IGR_226_2119730_2120188.sto.r2r

5′-AUYUAUAU●AGU●CCUC●UGGAAAAA●●G●●●●●●●GURYG●RARCUGGC●●RAAYYYUR●GGGYA●R●RA●CAUR●GRUUUUAC●GA●C●RR●●UGY●GRA●AA●●Y●●GARRY●CY●CC●GAAA●GU●YYCYGA●RRAUU●RAU●●○○○
